# Insight on Transcriptional Regulation of the Energy Sensing AMPK and Biosynthetic mTOR Pathway Genes

**DOI:** 10.3389/fcell.2020.00671

**Published:** 2020-07-29

**Authors:** Abitha Sukumaran, Kwangmin Choi, Biplab Dasgupta

**Affiliations:** ^1^Division of Oncology, Cincinnati Children’s Hospital Medical Center, University of Cincinnati College of Medicine, Cincinnati, OH, United States; ^2^Division of Experimental Hematology and Cancer Biology, Cincinnati Children’s Hospital Medical Center, University of Cincinnati College of Medicine, Cincinnati, OH, United States

**Keywords:** AMPK, mTOR, transcription, signaling, metabolism

## Abstract

The Adenosine Monophosphate-activated Protein Kinase (AMPK) and the Mechanistic Target of Rapamycin (mTOR) are two evolutionarily conserved kinases that together regulate nearly every aspect of cellular and systemic metabolism. These two kinases sense cellular energy and nutrient levels that in turn are determined by environmental nutrient availability. Because AMPK and mTOR are kinases, the large majority of studies remained focused on downstream substrate phosphorylation by these two proteins, and how AMPK and mTOR regulate signaling and metabolism in normal and disease physiology through phosphorylation of their substrates. Compared to the wealth of information known about the signaling and metabolic pathways modulated by these two kinases, much less is known about how the transcription of AMPK and mTOR pathway genes themselves are regulated, and the extent to which AMPK and mTOR regulate gene expression to cause durable changes in phenotype. Acute modification of cellular systems can be achieved through phosphorylation, however, induction of chronic changes requires modulation of gene expression. In this review we will assemble evidence from published studies on transcriptional regulation by AMPK and mTOR and discuss about the putative transcription factors that regulate expression of AMPK and mTOR complex genes.

## Introduction

Adenosine Monophosphate-activated Protein Kinase (AMPK) is a serine-threonine kinase that exist as a heterotrimer of catalytic α and regulatory β and γ subunits (Davies et al., 1994; Mitchelhill et al., 1994). Mammals express two catalytic α1 and α2 subunits, two regulatory β1 and β2 subunits and three additional nucleotide-binding regulatory γ1, γ2, and γ3 subunits genes (Viollet et al., 2010; Carling et al., 2012; Hardie, 2014a). The N-terminus of the α subunits contain the catalytic domain as well as a phosphorylation site for upstream kinases that regulate its activity (Crute et al., 1998). The γ subunits bind to adenosine monophosphate/adenosine diphosphate AMP/ADP and play a regulatory role, while the conserved C-terminus of the β subunit interacts with the α and γ subunits and is required for AMPK complex formation (Spasic et al., 2008; Dasgupta and Milbrandt, 2009). The seven AMPK subunits are expressed more or less ubiquitously. However, each of the twelve possible αβγ AMPK complex display considerable variation in tissue-specific expression, subunit association, subcellular localization and function (Quentin et al., 2011; Dasgupta et al., 2012). Two upstream kinases – liver kinase B1 (LKB1) Serine/threonine kinase 11 (STK11) and calcium calmodulin-dependent protein kinase kinase β (CaMKKβ) phosphorylate the γ subunits to fully activate AMPK (Hawley et al., 2003, 2005; Shaw et al., 2004; Hurley et al., 2005; Oakhill et al., 2010, 2011; Xiao et al., 2011). LKB1 exists in a ternary complex with STRAD (STE20-related adaptor) and CAB39/MO25 (mouse protein 25), and LKB1 activity in the complex is 10-fold higher than LKB1 alone (Baas et al., 2003). While LKB1 activates AMPK in response to AMP/ADP, CaMKKβ activates AMPK in response to Ca^2+^. Both pathways can act in isolation or synergistically (Woods et al., 2005). The interaction of LKB1 with the AMPK complex has been shown to be facilitated by the cytoplasmic protein AXIN (Zhang et al., 2013), which also interacts with numerous other proteins. Needless to say that transcriptional regulation of the two upstream AMPK kinases could also determine AMPK activation.

The mTOR kinase exists is two distinct complexes – mTORC1 and mTORC2. The C1 complex is composed of five proteins – mTOR kinase, RAPTOR (regulatory-associated protein of mTOR), mLST8 (mammalian lethal with Sec13 protein 80), PRAS40 (proline-rich AKT substrate 40 kDa), and Deptor (Dep-domain containing mTOR-interacting protein). The C2 complex is composed of six proteins – mTOR kinase, RICTOR (rapamycin-insensitive companion of mTOR), mSIN1 (mammalian stress-activated protein kinase interacting protein), Protor-1 (protein observed with Rictor-1), mLST8 and Deptor (Laplante and Sabatini, 2009, 2013; Saxton and Sabatini, 2017). In some tissues, a negative feedback from mTORC1 controls mTORC2 such that mTORC1 activation reduces mTORC2 activity.

Excellent reviews have been written on the signaling mechanisms regulated by AMPK and mTOR (Hardie, 2014b; Hardie et al., 2016; Carling, 2017; Garcia and Shaw, 2017). Therefore, we will not elaborate on this topic here. Instead, we will emphasize on the transcriptional regulation of the AMPK –mTOR pathway genes and how these two pathways regulate gene expression, beyond signaling.

## AMPK and mTOR Pathway Genes

The genes that encode the seven subunits of AMPK in mammals are *Prkaa1* (α1), and *Prkaa2* (α2), *Prkab1* (β1), *Prkab2* (β2), *Prkag1* (γ1), *Prkag2* (γ2), and *Prkag3* (γ3) (Viollet et al., 2010; Carling et al., 2012; Hardie, 2014a). The rooted trees of the α and β subunits suggest that vertebrate *Prkaa1/a2* and *Prkab1/b2* genes arose by duplications of ancestral genes in lower eukaryotes (Rider, 2016). With one known exception, homologs of AMPK subunits are present in all living organisms indicating that AMPK subunits were selected early in evolution (Lin and Hardie, 2018). AMPK subunits have been reported in the fruit fly *Drosophila melanogaster*, the nematode *Caenorhabditis elegans*, the budding yeast *Saccharomyces cerevisiae*, the flowering plant *Arabidopsis Thaliana* and other plants (Polge and Thomas, 2007; Emanuelle et al., 2015), and the primitive protozoon *Giardia lamblia*. The lone exception known so far is the unicellular eukaryotic microsporidian *Cephalitozoon cuniculi* whose genome does not seem to encode any *Prkaa* AMPK gene (Katinka et al., 2001; Miranda-Saavedra et al., 2007). The *Drosophila* genome encodes three AMPK subunits in total – *Dmel/Ampka*, *Dmel/alc* (Alicorn or *Ampkb*), and *Dmel/SNF4Ag* (FlyBase). *C. elegans* express two catalytic α subunits (*Aak1* and *Aak2*), two β subunits (*Aakb1* and *Aakb2*), and five putative γ subunits (*Aakg1-5*) (WormBase version *Ws238*). In the budding yeast AMPK subunits are encoded by five genes - *Snf1a* (α subunit), *Sip1*, *Sip2*, and *Gal83b* (β subunits) and *Snf4g* (γ subunit) (Hedbacker and Carlson, 2008). In *Arabidopsis* the SNF1-related kinase 1 (*Snrk1*), a homolog of yeast *Snf1* and mammalian *Prka* are transcribed by two typical α subunits (*Kin10* and *Kin11*), two β subunits, one γ subunit along with two atypical subunits - β3 and βγ (Emanuelle et al., 2015). In addition to the AMPK holoenzyme, upstream kinases that are required for full activation of AMPK are also preserved across species. In mammals these upstream kinase genes are *Stk11* (Lkb1) and *Camkk2*. Their homologs in lower eukaryotes include *Par4* in *C elegans*, *Dmel/Lkb1* in *D. melanogaster*, *Pak1*, *Tos3*, and *Elm1* in *S. cerevisiae*, and *Grik1* and *Grik2* in *A. thaliana* (Hawley et al., 2003; Hong et al., 2003; Shaw et al., 2004; Shen et al., 2009).

Similar to AMPK which is a multimeric complex, the mTORC1 complex contains five proteins in mammals. The genes encoding these five subunits are *Mtor*, *Raptor*, *Deptor*, *Lst8*, and *Akt1s1* (PRAS40). The mTORC2 complex which is composed of six proteins are encoded by *Mtor*, *Rictor*, *Sin1*, *Lst8*, *Prr5l* (PROTOR2), and *Deptor*. Akin to mammals, *S. Cerevisiae* also have TORC1 and TORC2 complexes (Loewith and Hall, 2011) including homologs of *Raptor* (*Kog1*), *mLST8* (*Lst8*), *Rictor* (*Avo3*), and *mSin1* (*Avo1*), although additional components are specific to yeast or mammals (Saxton and Sabatini, 2017). In *C elegans* and *D. melanogaster*, the C1 and C2 genes are encoded by *Tor*, *Raptor*, *Lst8*, *Rictor*, and *Sin1*. Beyond this, there appears to be a degree of unexplained incongruity among organisms in the evolution of the mTOR complex genes. While the protist *Dictyostelium discoideum* (unicellular common slime mold, a species of soil-dwelling amoeba) encodes all five mTORC1/C2 genes, all plants encode the *Tor*, *Raptor*, *Lst8* genes. Other unicellular organisms such as the diatom *Phaeodactylum tricornutum* and the intestinal parasite *Giardia intestinalis* encode only mTORC1 genes, while the free-living ciliates such as *Tetrahymena thermophila* encodes only TORC2 genes. Breaking the rule among lower organisms, the free-living unicellular protist *Naegleria gruberi* and the human parasite *Leishmania major* express genes for both TORC1 and C2 complex. The malarial parasite *Plasmodium falciparum* seems to be an exception that does not encode any mTOR complex genes (van Dam et al., 2011). Thus, it appears that while some lower organisms and plants have adapted and evolved successfully with just one TOR complex, perhaps specific nutritional control mechanisms and environmental pressures necessitated the presence of both mTORC1 and C2 genes in other organisms.

## The AMPK-mTORC1 Signaling Axis

Mechanistic Target of Rapamycin1 is an anabolic kinase essential for the biosynthesis of key macromolecules such as protein, lipid and nucleotides (Laplante and Sabatini, 2009; Hindupur et al., 2015; Saxton and Sabatini, 2017). AMPK on the other hand functions to check mTORC1 activity when energy and raw materials for macromolecule production becomes limiting, and as a catabolic enzyme to simultaneously augment glucose import and energy production through glycolysis and mitochondrial oxidative phosphorylation (Mihaylova and Shaw, 2011; Hardie, 2014a; Dasgupta and Chhipa, 2016).

Adenosine Monophosphate-activated Protein Kinase downregulates mTORC1 by two independent mechanisms - through phosphorylation of the tumor suppressor Tuberous Sclerosis Complex 2 (TSC2) and RAPTOR. Extensive literature exists on this area (Laplante and Sabatini, 2009, 2013; Mihaylova and Shaw, 2011; Hindupur et al., 2015; Dasgupta and Chhipa, 2016; Saxton and Sabatini, 2017; Herzig and Shaw, 2018), and therefore the signaling axis will only be outlined here. Growth factor signaling loads GTP to the small GTPase called Rheb (Ras homolog enriched in brain), that activates mTORC1 on the lysosomal surface. TSC which is a GTPase activating protein (GAP) for Rheb, converts Rheb-GTP to Rheb-GDP, and thereby inhibits mTORC1 activity (Saxton and Sabatini, 2017). Upstream of TSC, growth factor signaling through Akt, Erk and other kinases phosphorylate TSC to inhibit its GAP activity, and thus enabling mTORC1 activation. On the other hand, under various conditions including energy stress, AMPK phosphorylates TSC2 to enhance its GAP activity causing mTORC1 inhibition (Inoki et al., 2003). While growth factor signaling-induced mTORC1 activation is controlled by AMPK-TSC2 interaction, amino acid-induced mTORC1 activation is controlled by AMPK-RAPTOR interaction (Gwinn et al., 2008). Amino acid adequacy allows activation of the RAG family of small GTPases which binds to RAPTOR and recruits mTORC1 to the lysosome for further activation by Rheb-GTP (Kim et al., 2008; Sancak et al., 2008). During energy stress, AMPK phosphorylates RAPTOR to directly inhibit RAPTOR-mTOR interaction and mTORC1 activation. Although AMPK is localized in nucleus or cytoplasm under various physiological stress, a recent study showed its presence in late endosomes/lysosomes, suggesting that lysosomes serve as a site for AMPK/mTORC1 signaling (Zhang et al., 2014). The preferential requirement of TSC2 versus RAPTOR for mTORC1 regulation under various physiological and pathological contexts remains to be fully understood.

## Protein Abundance as a Determinant of Kinase Function

Protein phosphorylation by kinases is one of the most widely studied post-translational modifications (PTM) (Levy et al., 2012). While significant effort has been given to identify phosphosites on kinase substrates and investigate the functional consequences of site-specific phosphorylation, the abundance and stoichiometry of kinases and substrates as functions of proteins are less appreciated (Levy et al., 2012). Although substrate phosphorylation is a regulated process where substrates, kinases and phosphatases are organized via adaptor and scaffold proteins (Bhattacharyya et al., 2006; Scott and Pawson, 2009), a more abundant kinase or a substrate are indeed more likely to come across each other than less abundant proteins (Lienhard, 2008). On the other hand, less abundant proteins can be focally concentrated on the surface of cytosolic organelles such as lysosomes or the endoplasmic reticulum to achieve high level of activity. Kinase-substrate stoichiometry is also crucial – the probability of an abundant substrate to be optimally phosphorylated becomes low if its kinase is suboptimally expressed (Wu et al., 2011; Levy et al., 2012). Although protein abundance at a given time can be regulated at multiple levels such as translation efficiency and turnover rates, transcription rate is also likely to be a key determining factor (Vogel and Marcotte, 2012; Li et al., 2014; Liu et al., 2016). Is transcription of kinases and its context-specific substrates coordinated? How a kinase and its specific substrates are made available in sufficient and proportional quantities in response to an environmental input? To the best of our knowledge, there are no in-depth studies investigating whether and how subunit abundance of AMPK and mTOR complexes are regulated in cell-type specific and context dependent manner, and how that impacts the inhibitory effect of AMPK on mTORC1. Transcription is an highly regulated and complex process with TFs binding up to several kilobases upstream and downstream of the transcription start site of genes, and co-activators and co-repressors binding to distant enhancers (sometimes located on different chromosomes) that loop in to modulate transcription efficiency (Chen and Rajewsky, 2007). Inclusion of all these factors to determine how TFs self-organize and identify binding sites to regulate transcription is not only a formidable task but would require a comprehensive review on this topic itself. Instead, in the next section, we discuss about the transcription factors that bind to *cis* regulatory elements proximal to transcription start sites of the AMPK and mTOR pathway genes.

## Transcription Factors Regulating AMPK and mTOR Pathway Gene Expression

**FIGURE 1:**
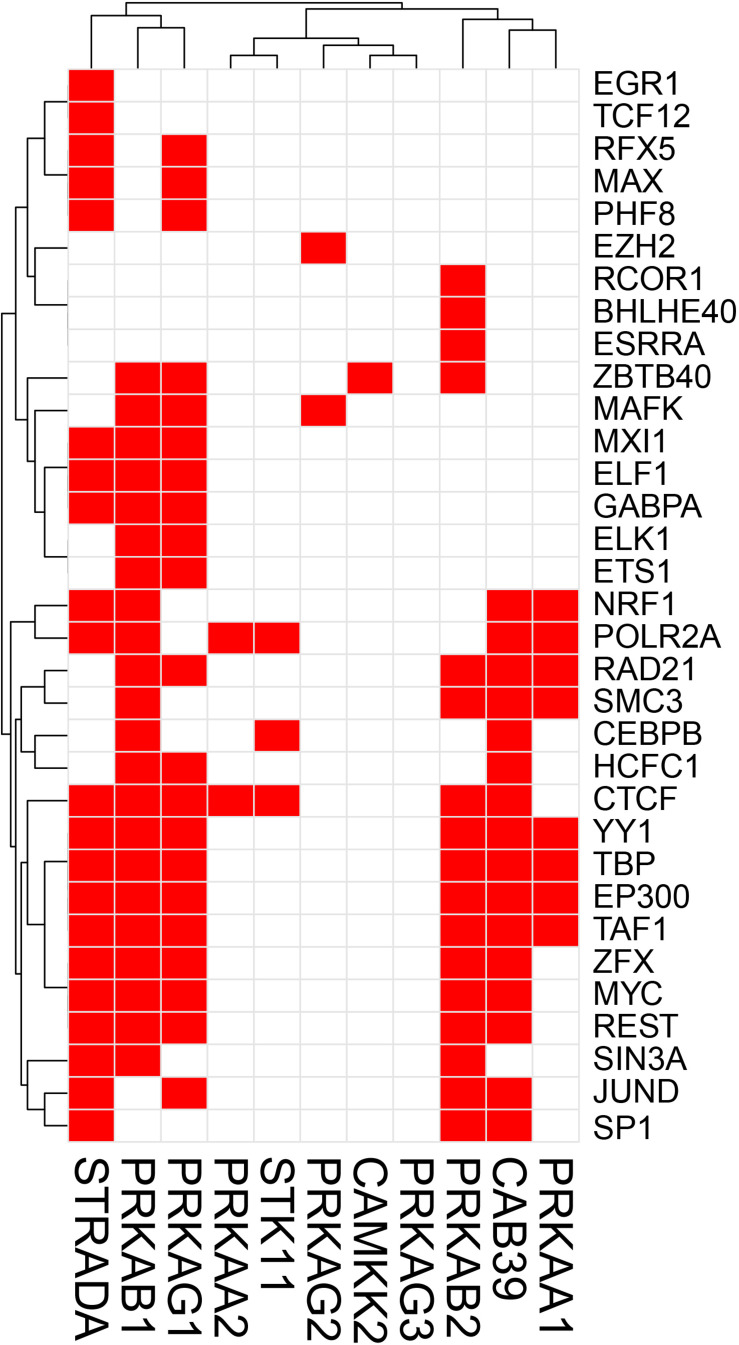
Heatmap-style “occurrence” plots of putative AMPK family TFs. The occurrence plot is an intuitive way to visualize co-regulated genes whose expressions are controlled by the same TF(s). Briefly, we assigned “1” if the binding site(s) of a given TF’s (right) are reported “at least once” (in ENCODE) within a reasonable distance from the transcription start site (TSS) of a gene (bottom). Otherwise, we assigned “0” (i.e., no TFBS within a given range). The result is a matrix filled with 0 (w/o TFBS) and 1 (w/TFBS). The trees on the top and the left sides were generated using the hierarchical clustering based on vectors in rows and columns. AMPK pathway genes and TF-binding peak occurrence are shown in the promoter regions. Each red cell represents a gene with at least one binding sites of a given TF within 2 kb-upstream and 1 kb-downstream from its TSS.

**FIGURE 2:**
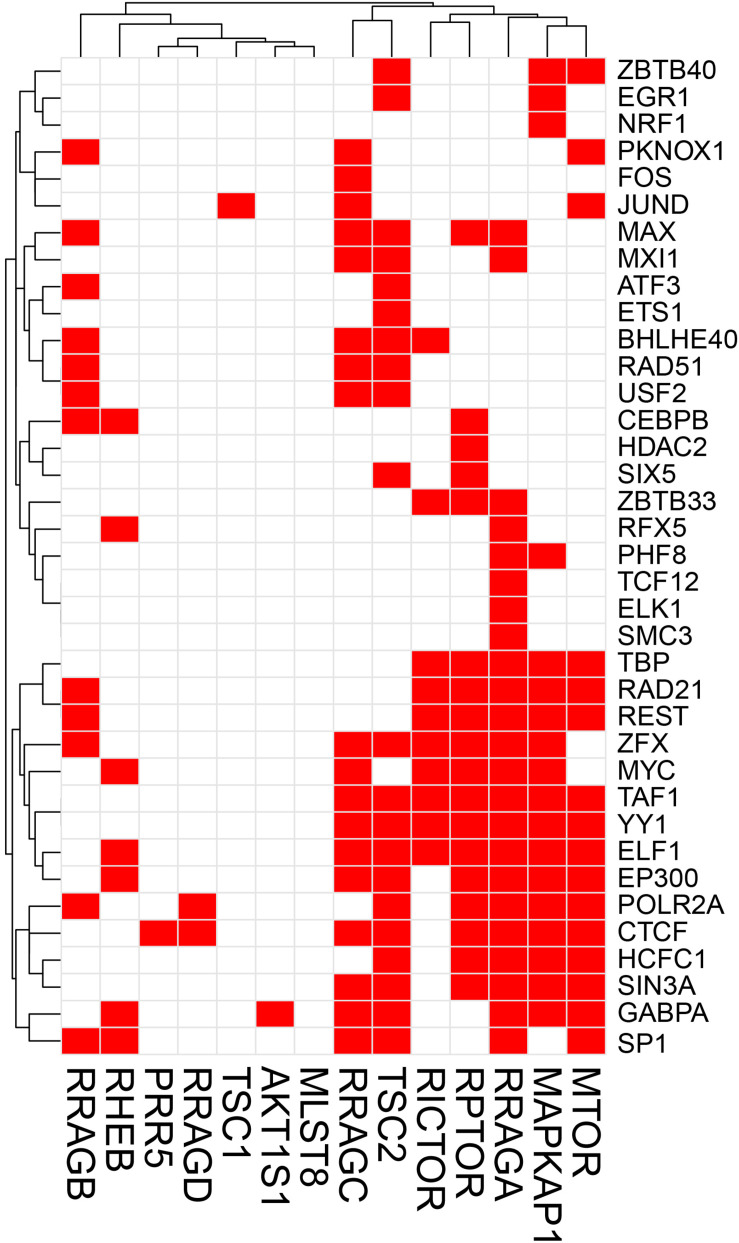
Heatmap-style “occurrence” plots of putative mTOR family TFs. The occurrence plot is an intuitive way to visualize co-regulated genes whose expressions are controlled by the same TF(s). Briefly, we assigned “1” if the binding site(s) of a given TF’s (right) are reported “at least once” (in ENCODE) within a reasonable distance from the transcription start site (TSS) of a gene (bottom). Otherwise, we assigned “0” (i.e., no TFBS within a given range). The result is a matrix filled with 0 (w/o TFBS) and 1 (w/TFBS). The trees on the top and the left sides were generated using the hierarchical clustering based on vectors in rows and columns. mTOR pathway genes and TF-binding peak occurrence are shown in the promoter regions. Each red cell represents a gene with at least one binding sites of a given TF within 2 kb-upstream and 1 kb-downstream from its TSS.

The potential TF binding sites (TFBSs) can be predicted using the known and inferred motifs, represented as position-specific scoring matrices (PSSMs) derived from the various binding models such as protein-binding microarrays (PBMs), high-throughput SELEX (HT-SELEX), and manually curated models including JASPAR^1^ and/or TRANFAC^2^ (Orenstein and Shamir, 2014; Hombach et al., 2016). Due to the presence of multiple TFs in more than one binding model source, computationally predicted sites can include many false hits. We therefore collected human TF-binding locations from the ENCODE ChIP-seq hg38 dataset (v3)^3^. This dataset contains “*in vivo*” TF binding peaks from 1256 experiments representing 340 TFs in 129 cell types. For the sake of simplicity, we have only considered peaks supported by at least 4 cell types and found within the 2 Kb upstream and the 1 Kb downstream regions of the start site where most of the TF binding sites (TFBS) are located. We would like to recognize that although in most TF binding site (TFBS) distribution studies, the most relevant TFBSs are generally located ±2 Kb from the TF start site, more complex regulatory circuits may be constructed which are beyond the scope of this review. ChIP-seq has been instrumental in the examination of TF-binding sites (Johnson et al., 2007). Caveats about using ChIP-seq data are that the method can clearly pick up indirect interactions (e.g., the ChIP antibody binds to a TF which instead of directly interacting with DNA interacts to another DNA-bound TF), and many antibodies are not ChIP-grade and can cross-react. Another limitation of ENCODE or similar databases such as ChIP-ATLAS, Cistrome, and ReMap-ChIP is that all these databases are built upon the availability of ChIP-grade antibodies, and therefore several genuine TFs could be missed for which tools are not yet available. Notwithstanding these cautions, we examined ENCODE ChIP-seq data to identify potential TFs for the following genes: *Prkaa1*, *Prkaa2*, *Prkab1*, *Prkab2*, *Prkag1*, *Prkag2*, *Prkag3*, *Stk11*, *Strada*, *Cab39* (MO25), *Camkk2* in the AMPK pathway, and *Tsc1*, *Tsc2*, *Rptor*, *Rictor*, *Mtor*, *Mlst8*, *Akt1s1* (PRAS40), *Mapkap1* (SIN1), *Prr5* (PROTOR), *Rheb*, *Rraga* (RAGA), *Rragb* (RAGB), *Ragc* (RAGC), and *Ragd* (RAGD) in the mTOR pathway. This analysis revealed that *Prkab1*, *Prkab2*, *Prkag1*, and the *Stk11* (LKB1) binding partners *Cab39* and *Strada* in the AMPK pathway (Figure 1), and *Mtor*, *Mapkap1*, *Rraga*, *Rragc*, *Rptor*, and *Tsc2* in the mTOR pathway (Figure 2) have the highest number (10 or more) of TFs. On the contrary, *Prkaa2*, *Prkag2*, *Prkag3*, *Stk11*, and *Camkk2* in the AMPK pathway (Figure 1), and *Akt1s1*, *Tsc1*, *Rragd* and *Prr5* in the mTOR pathway (Figure 2) are transcribed by the fewest number (1–4) of TFs. The genes with the fewest number (only 1) of assigned TFs were *Camkk2*, *Akt1s1*, *Tsc1*, while the genes with the highest number (21–23) of assigned TFs were *Prkag1*, *Prkab1*, *Strada*, *Rraga* and *Tsc2*.

A simple explanation of these results is that transcriptional regulation of some genes of the AMPK and mTOR pathways are more flexible and more adaptive with several TFs able to initiate transcription under various cues, while regulation of other genes with just one or two TFs is tight and critically depend on the abundance of these TFs. An alternative explanation is that one or two TFs are sufficient for optimal transcription of some genes, while other genes require cooperative interaction of multiple TFs for optimal transcription. It is also possible that the one or two TFs that we identified for some genes in the 2 Kb upstream and 1 Kb downstream region of the TSS are actually insufficient and require coordination of co-activators and distant enhancers. Another intriguing observation that is apparent from Figures 1, 2 is that there seems to be a cluster of common TFs that regulate multiple genes of both AMPK and mTOR pathway. For example, the TFs YY1, TBP, EP300, RAD21, REST, Myc, SIN3A, CTCF, and TAF1 has binding sites in half of the twenty-five AMPK and mTOR pathway genes. We speculate that environmental cues probably converge on common TFs to regulate transcription of AMPK and mTOR pathway genes in a coordinated fashion such that the required abundance and stoichiometry is maintained for optimal signaling output.

## AMPK May Control Gene Expression Through Metabolites of Intermediary Metabolism

**FIGURE 3:**
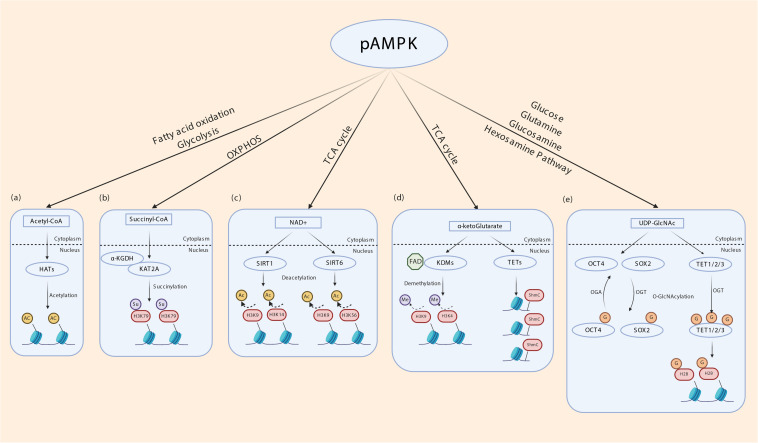
Indirect transcriptional control by AMPK through metabolites of intermediary metabolism. (a) Intermediary metabolism of glucose, fatty acids and ketogenic amino acids leads to the formation of acetyl-CoA. By regulating glycolysis and fatty acid oxidation AMPK may determine cellular acetyl-CoA levels. Histone acetyltransferases (HATs) use acetyl-CoA to transfer acetyl group to nucleosomal histones. (b) α ketoglutarate dehydrogenase subunit E2 (α-KGDH) complex binds to lysine acetyltransferase 2A (KAT2A). α-KGDH synthesizes succinyl CoA locally and KAT2A succinylates histone H3 on lysine 79 (H3K79). AMPK regulates TCA cycle thereby may influence this process. (c) NAD generated in the mitochondrial electron transport chain acts as a cofactor for SIRT1 and SIRT6 which deacetylates histone H3K9/14 and H3K9/56, respectively. (d) α-ketoglutarate (α-KG), generated through TCA cycle acts as a cofactor for lysine demethylases (KDM) and ten-eleven translocation (TET) enzymes. TETs oxidize 5-methyl-2′-deoxycytidine in genomic DNA to 5-hydroxymethylcytosine (5hmC), 5-carboxylcytosine (5caC), and 5-formylcytosine (5fC) that is involved in epigenetic regulation. FAD generated in the mitochondrial electron transport chain acts as a cofactor for KDMs. (e) AMPK potentially regulates the hexosamine pathway by providing precursors. UDP-GlcNAc is derived from glycolysis and glutamine, the latter being generated by transamination of α ketoglutarate. O-GlcNAcyltransferase (OGT) transfers GlcNAc residues to various nuclear proteins including TETs and histone 2B (H2B), OCT4 and SOX2 to control transcription.

It is now well-established that both metabolic enzymes and metabolites synthesized in intermediary metabolism directly or indirectly impact gene expression. DNA itself and DNA-binding histones are modified and regulated by several mechanisms that uses metabolites generated in intermediary metabolism (Etchegaray and Mostoslavsky, 2016; van der Knaap and Verrijzer, 2016; Nieborak and Schneider, 2018). These include nicotinamide adenine dinucleotide (NAD) (used in SIRT mediated histone deacetylation and histone polyADP-ribosylation) (Vaquero et al., 2006; Michishita et al., 2008; Kawahara et al., 2009; Nakahata et al., 2009; Zhong et al., 2010; Barber et al., 2012; Etchegaray et al., 2013; Imai and Guarente, 2014), flavin adenine dinucleotide FAD (used in lysine-specific histone demethylation by LSD1/2) (Shi et al., 2004; Shi and Whetstine, 2007; Ciccone et al., 2009; Karytinos et al., 2009; Dimitrova et al., 2015), acetyl co-A (used in histone acetylation), S-adenosylhomocysteine (SAM, used in DNA methylation) (Takusagawa et al., 1996; Mentch et al., 2015), α-ketoglutarate (used to regulate DNA methylation through activation of the TET and JMJD family of DNA demethylases) (Xiao et al., 2012; Pastor et al., 2013), succinyl CoA (used in succinylation of the histone acyltransferase KAT2A) (Wang et al., 2017), UDP-GlcNAc (used O-GlcNAcylation of histones) (Sakabe et al., 2010; Fujiki et al., 2011; Nardini et al., 2013; Lewis and Hanover, 2014), among others. Therefore, the activity of metabolic enzymes that regulate intermediary metabolic cycles in essence controls DNA and histone modifications and thereby gene expression. Besides generating ATP from glucose oxidation to maintain energy homeostasis, AMPK enhances glycolysis and glucose oxidation through the TCA cycle and mitochondrial oxidative phosphorylation by various mechanisms. It increases glucose transporter expression and cell surface translocation (Kurth-Kraczek et al., 1999; Xi et al., 2001; Kishton et al., 2016; Siques et al., 2018), activates phosphofructokinase 2 (PFK2) (Marsin et al., 2000), and cAMP Responsive Element Binding Protein 1 (CREB1) (Chhipa et al., 2018), and transcriptionally upregulates Peroxisome proliferator-activated receptor gamma coactivator 1-alpha (PGC1α) (Terada et al., 2002; Suwa et al., 2003). NAD is generated from pyruvate especially in highly glycolytic lactate producing cells, while both FAD and NAD are produced in the mitochondrial electron transport chain complex I and II (Rogatzki et al., 2015). Acetyl co-A, α ketoglutarate and succinyl CoA are all produced in the TCA cycle, and UDP-GlcNAc is produced in the hexosamine pathway that originates from glucose phosphorylation in glycolysis (Jeremy et al., 2002). Therefore, environmental cues that activate AMPK can in turn regulate gene expression through glucose metabolism-derived metabolites (Figure 3).

Glucose metabolism impacts gene expression. At the systemic level, glucose stimulates insulin expression in the pancreas (Schuit et al., 1988; Rorsman and Braun, 2013). In the liver, glucose induces glucose transporter expression, glycolytic and lipogenic enzymes (Rui, 2014; Han et al., 2016), and in the muscle it induces glucose transporters for insulin-induced glucose uptake (Klip and Paquet, 1990; Saltiel and Kahn, 2001). However, glucose is metabolized by every cell outside these three primary metabolic organs. What is the impact of glucose on gene expression at the cellular level outside the primary metabolic organs? The effects of AMPK on glucose metabolism-dependent gene expression could be acute or chronic. In the large majority of published studies, this effect of AMPK has been studied in glucose-starved cells that were acutely re-exposed to glucose *in vitro*, an experimental condition that reduces AMPK activity in most if not all normal cells. In several cancer cells, however, high basal AMPK activity is insensitive to such manipulation of glucose levels (Chhipa et al., 2018). While acute exposure of glucose-starved cells depresses AMPK activation, active AMPK levels quickly reach steady state levels, sometimes in minutes. It is imperative to understand that following glucose addition, because of the steady flux of glucose import and consumption through glucose phosphorylation, intracellular glucose levels are not reduced significantly by the time steady state levels of active AMPK levels have been reached. What is the need for AMPK activity to resurge so quickly when the cells are actively metabolizing glucose? And what role active AMPK plays during steady state cellular glucose metabolism in normal and tumor cells? It will be interesting to learn if glucose metabolism-dependent global gene expression is affected in the absence of AMPK. If AMPK is required for the optimal generation of chromatin modifying metabolites in glycolysis and TCA cycle, the prediction is that global gene expression will be significantly altered in AMPK null cells.

Recent reports demonstrating the involvement of AMPK in regulating the tumor suppressor gene folliculin (FLCN) came from Collodet et al. (2019). In this study, the authors have shown that AMPK induces FLCN expression via the transcription factors transcription factor EB (TFEB) and transcription factor binding to IGHM enhancer 3 (TFE3) independent of mTOR. This is one of the few reports that demonstrate the mTOR-independent effects of AMPK on the transcription factors TFEB and TFE3. mTOR independent effects of AMPK on these TFs was also recently reported by El-Houjeiri et al. (2019) in which the authors showed that TFEB and TFE3 regulate innate immune response via AMPK/FLCN signaling axis. The role of AMPK in regulating TFEB levels was also shown by Young et al., in mouse embryonic stem cells (ESCs). ESCs lacking AMPK were normal in the pluripotent state, but developed profound defects during differentiation. TFEB was found to be the most significantly downregulated gene in AMPK deficient cells leading to reduced endolysosomal activity. Similar defects were seen in TFEB null ESCs linking TFEB and diminished lysosomal activity to germ cell specification (Young et al., 2016).

## mTORC1 Regulation of Transcription

**FIGURE 4:**
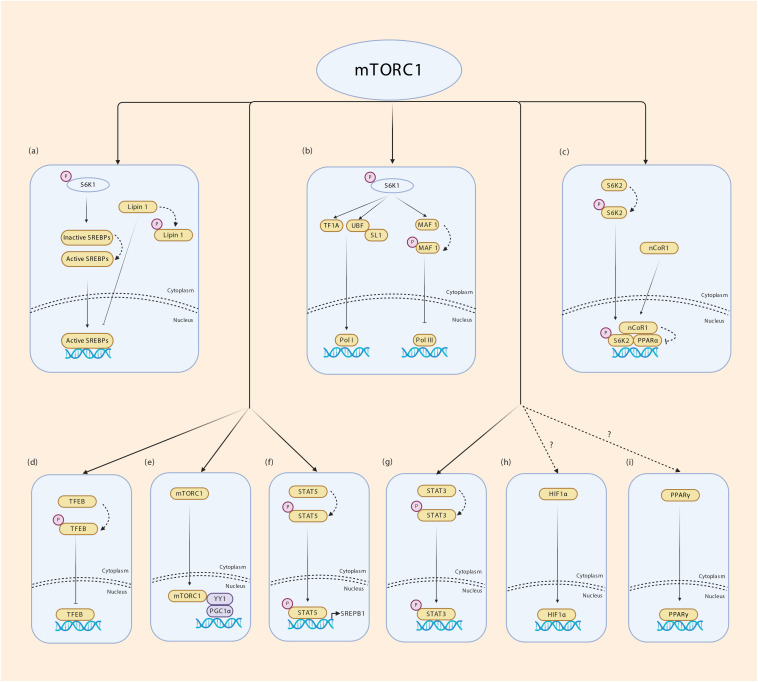
Transcriptional regulation by mTORC1. (a) mTORC1 regulates fatty acid synthesis by enabling SREBP1 mediated transcription. Lipin 1 inhibits SREBPs from binding to their target genes. mTORC1 phosphorylates Lipin 1 and prevents its translocation to the nucleus. S6K1 phosphorylation by mTORC1 is required for cleavage and activation of SREBP1c through a yet unknown mechanism. (b) mTORC1 phosphorylates S6K1 and activates tripartite motif-containing protein-24 (TIF24) and promotes its interaction with Pol I. Phosphorylated S6K1 also promotes the interaction of upstream binding factor (UBF) with SL1 regulating rRNA expression. mTORC1 phosphorylates MAF 1 which is a repressor of Pol III thereby controlling the expression of 5srRNA and tRNA. (c) mTORC1 phosphorylates S6K2 which interacts with nuclear receptor corepressor 1 (nCoR1) promoting its translocation to the nucleus thereby inhibiting PPARα. (d) mTORC1 phosphorylates TFEB in the surface of lysosomes promoting its binding to 14-3-3 proteins inhibiting its transport into nucleus. (e) Nuclear mTORC1 interacts with YY1 which in turn modulates the transcriptional activity of PGC1α. (f) mTORC1 phosphorylates STAT5 and promotes SREBP1 transcriptional activity. (g) mTORC1 phosphorylates STAT3 allowing transcription of STAT3 target genes. (h,i) mTORC1 regulates HIF1α and PPARƔ transcription through unknown mechanisms.

Many TFs require post-translational modifications (PTM) for activity, nuclear/cytoplasmic translocation, interaction with binding partners including other TFs, stability and DNA binding (Benayoun and Veitia, 2009; Filtz et al., 2014). These include phosphorylation, glycosylation, methylation, acetylation, sumoylation and ubiquitination. Phosphorylation is a straight forward and reversible modification that transmits signal from extracellular cues through growth factor signaling to TF activity. Phosphorylation can also cooperate with or antagonize other PTM to modulate TF activity. Several TFs have been shown to be regulated by mTORC1 – this includes yin yang 1 (YY1), sterol regulatory element binding proteins SREBPs, signal transducer and activator of transcription STAT3, PGC1α, hypoxia inducible factor 1 alpha (HIF1α), peroxisome proliferator-activated receptors PPARγ/α and TFEB (Laplante and Sabatini, 2013). Nuclear Lipin 1 binds to SREBPs and impairs SREBP-mediated transcription of lipogenic genes. mTORC1 phosphorylates Lipin 1 to cause its nuclear exclusion, thus controlling lipogenic gene expression (Laplante and Sabatini, 2013). In response to excess amino acids or ciliary neurotrophic factor (CNTF) signaling, mTORC1 directly phosphorylates STAT3 at S727 to promote its transcriptional activity (Yokogami et al., 2000; Kim et al., 2009). The SREBP family of bHLH TFs regulate lipogenesis. Multiple studies have shown that mTORC1 increases lipogenesis by augmenting SREBP1 mRNA and protein levels, processing and nuclear enrichment (Li et al., 2010, 2011; Wang et al., 2011; Yecies et al., 2011; Bakan and Laplante, 2012; Owen et al., 2012). A recent study by Li et al. (2019) has identified STAT5 phosphorylation by mTORC1 in promotion of SREBP1 transcriptional activity, thereby establishing a molecular mechanism by which mTORC1 controls SREBP1. The role of mTORC1 in PPARγ-mediated adipogenesis is controversial. While some studies show a positive effect on adipogenesis, others show a negative effect of mTORC1 on adipogenesis (Kim and Chen, 2004; Polak et al., 2008; Laplante and Sabatini, 2012). Through its downstream kinase S6K2, mTORC1 inhibits PPARα activity and PPARα- mediated hepatic ketogenesis (Sengupta et al., 2010; Kim et al., 2012), however, evidence for a direct phosphorylation event in this phenomenon is lacking. During hypoxia, mTORC1 possibly increases HIF1α transcription although mechanisms are unknown (Laughner et al., 2001; Hudson et al., 2002; Brugarolas et al., 2003; Duvel et al., 2010). Lysosomes that act as scaffold for AMPK-mTORC1 interaction responds to environmental signals and take part in stress response during which rapid expansion of lysosomal membrane takes place. mTORC1 phosphorylates TFEB which triggers 14-3-3 binding and cytoplasmic sequestration of TFEB. During nutrient stress which inhibits mTORC1, TFEB is released, translocates to the nucleus to coordinate lysosomal membrane synthesis (Martina et al., 2012; Roczniak-Ferguson et al., 2012; Settembre et al., 2012). mTORC1 was also reported to orchestrate mitochondrial biogenesis through its interaction with PGC1α and YYI (Cunningham et al., 2007; Koyanagi et al., 2011). It is well known that mTORC1 is present in the nucleus. However, how mTORC1 interacts with PGC1α and YYI to coordinate mitochondrial biogenesis remains unclear. mTOR regulation of transcription is illustrated in Figure 4. Together, these studies indicate that although not all mechanisms are fully understood, the effect of mTORC1 on regulating TF activity is unequivocal.

## AMPK Regulation of Transcription: Role Beyond Signaling

### Transcriptional Regulation of Monosaccharide Sensing in Yeast and Mammalian Cells

Adenosine Monophosphate-activated Protein Kinase’s role in direct control of gene expression through TF regulation has been documented from the budding yeast *Saccharomyces* to mammalian cells. In the budding yeast *Saccharomyces cerevisiae*, glucose suppresses expression of a set of genes that are used to catabolize carbon sources other than glucose in glucose-limited growth conditions. Derepression/activation of glucose-repressed genes was found to rely on Snf1-dependent phosphorylation of multiple serine residues on the zinc finger TF Mig1 (Treitel et al., 1998; Smith et al., 1999). In contrast to yeast, mammalian cells heavily rely on glucose for rapid proliferation and survival. Some mammalian cells can sustain short periods of very low glucose levels in the presence of starch or maltose (Rheinwald and Green, 1974). However, glucose is the principle monosaccharide required for cell division and survival of mammalian cells. Growth of higher eukaryotic cells in secondary glucose sources in the normal physiological context is rare and therefore, whether AMPK plays an analogous role in glucose depleted conditions in higher eukaryotes is unknown. The mammalian ortholog of Mig1 are the Early Growth Response (EGR) family of TFs. It is, however, unknown if AMPK phosphorylates EGR family of TFs in mammalian cells.

### Transcriptional Control of Cell Cycle, Proliferation and Survival by AMPK

The role of AMPK in cell cycle control, proliferation, growth and survival by direct phosphorylation and transcriptional regulation of cell cycle proteins and TFs in the mammalian system is a fundamental yet understudied area in the AMPK field. Moreover, published results in this area need more confirmatory studies to understand the cell type and context-specific regulation of cell cycle and survival by AMPK. Studies in *Saccharomyces cerevisiae*, showed that the AMPK homolog Snf1 is required for transcription of genes for growth and survival in low glucose media or alternate carbon sources such as sucrose (Carlson, 1999). During inositol starvation, Snf1 phosphorylates histone 3 on S10 to allow recruitment of the acetyltransferase GCN5, DNA unfolding and transcription of INO1 transcription to promote survival (Lo et al., 2001). Histone phosphorylation by AMPK seems to be conserved in mammalian cells, since under various metabolic stresses, AMPK phosphorylated H2B on S36 to enhance transcription of survival genes (Bungard et al., 2010). Genetic studies in model organisms confirmed that loss of AMPK causes defects in cell polarity, growth and development of both early plants and animals (Lee et al., 2007; Mirouse et al., 2007; Bartkova et al., 2010; Wang and Kaufman, 2014). Active AMPK localized to the mitotic spindle of mammalian cells (Thaiparambil et al., 2012), although its significance is still unknown. Genome-wide survey of kinases required for mitosis, and genetic screen using AMPKα2 shRNA identified 28 novel proteins involved in chromosomal segregation, mitosis, cytokinesis and cytoskeletal reorganization that were phosphorylated by AMPK (Bettencourt-Dias et al., 2004; Fukuyama et al., 2007; Banko et al., 2011; Stenesen et al., 2013). AMPK was also found to be required for cell cycle progression of mammalian and *Drosophila* cells (Vazquez-Martin et al., 2009; Bartkova et al., 2010; Banko et al., 2011; Lu et al., 2011). About a decade ago, we reported that nuclear AMPK phosphorylates retinoblastoma protein (RB) to regulate G1-S transition of neural stem cells (Dasgupta and Milbrandt, 2009). RB phosphorylation by AMPK was independently confirmed in glioblastoma cells (Rios et al., 2013). Why a kinase that is known to generally counter growth would inhibit RB to allow proliferation? Whether this is true only in the neuroepithelial compartment or in other cell types outside the neuroepithelial compartment is yet to be determined. Intriguingly, AMPK was found to directly phosphorylate p53 on Ser15 to promote G1 arrest under nutrient stress. P53 is ubiquitinated and degraded to allow cell cycle progression, and its stabilization in dividing cells can lead to apoptosis or senescence (Lavin and Gueven, 2006). Another TF whose transcriptional activity is increased upon phosphorylation by AMPK is FoxO3 (Greer et al., 2007). FoxO family of TFs play important roles in growth factor signaling, proliferation, glucose metabolism and longevity (Lin et al., 1997; Ogg et al., 1997; Nakae et al., 2002; Giannakou et al., 2004; Hwangbo et al., 2004; Paik et al., 2007). AKT phosphorylates and sequesters FoxO family of TFs in the cytoplasm (Brunet et al., 1999; Kops et al., 1999; Tang et al., 2002). AMPK was shown to phosphorylate FoxO3 on six serine residues *in vitro* and in two residues (S413 and S588) in endogenous FoxO3 to enhance its transcriptional activity (Greer et al., 2007) toward its target genes including the cell cycle inhibitor p27 (Morgan, 1997; Medema et al., 2000). Therefore, it is conceivable that the contexts in which AMPK may phosphorylate RB, P53 or FoxO are discrete. The cell cycle machinery senses available cellular energy, and ATP depletion by as little as 15% has been found to cause G1 arrest, and a depletion by 35% caused G2M arrest of human promyelocytic leukemia cells (Sweet and Singh, 1995). It is not understood if AMPK’s role in mitosis is uncoupled from or linked to its energy sensing function. It is possible that basal nuclear AMPK activity phosphorylates a set of TFs to enable cell cycle proliferation when growth conditions are permissive, while a TF code switch during severely restrictive growth conditions signals cell cycle arrest to allow survival. This is an important yet unanswered question, and hopefully analysis of the nuclear phosphoproteomes integrated with single cell RNA-seq of proliferating cells will be able to address this issue.

### Transcriptional Control of Glucose and Mitochondrial Metabolism by AMPK

The requirement of AMPK for optimal glucose metabolism through glycolysis and oxidative phosphorylation has been reported in several cell culture and animal models. We have recently shown that AMPK phosphorylates the TF CREB1 on S133 to orchestrate transcription of HIF1α and GABPA, which are master regulators of the glycolysis and mitochondrial biogenesis transcriptional programs, respectively (Chhipa et al., 2018). CREB1 regulation by AMPK was also shown previously. In skeletal muscle, AMPK phosphorylates CREB1 on S133 to activate CREB1-dependent transcription. Interestingly, this is the same site targeted by PKA (Thomson et al., 2010). Thomson et al., showed that AMPK also phosphorylates other TFs such as CREM, ATF1 and CREBL2 in the skeletal muscle. CREB phosphorylation is not required for binding to CREB responsive elements, but it augments recruitment of coactivators such as CBP/p300 (De Cesare and Sassone-Corsi, 2000). Intriguingly, one study showed that AMPK can phosphorylate CBP/p300 at S89 to reduce its interaction with nuclear receptors such as PPARγ, thyroid receptor and retinoic acid receptors, but not with other TFs including CREB1, p53, E1A, and GATA1 (Yang et al., 2001). Reduced interaction of coactivators like CBP/P300 with PPARγ, seems at odds with the well-established evidence that AMPK promotes mitochondrial biogenesis and oxidative phosphorylation (OXPHOS) through activation of the PPARγ coactivator 1α (PGC1α) (Bergeron et al., 2001; Terada et al., 2002; Jorgensen et al., 2005; Reznick and Shulman, 2006; Garcia-Roves et al., 2008; Canto et al., 2010). The transcriptional coactivator PGC1α is a master regulator of mitochondrial biogenesis and OXPHOS. PGC1α activates two key nuclear respiratory factors NRF1 and NRF2 that activate the mitochondrial transcription factor TFAM which is essential for mitochondrial DNA replication and transcription of mitochondrial genes (Fisher et al., 1992; Virbasius and Scarpulla, 1994; Scarpulla, 2006). Several studies confirmed that AMPK enhances PGC1α expression and activity, and PGC1α - dependent mitochondrial gene expression (Suwa et al., 2003; Jorgensen et al., 2005; Jager et al., 2007; Irrcher et al., 2008; Canto et al., 2009), but the mechanisms remain contested. One study showed direct phosphorylation of PGC1α by AMPK on T177 and S538 (Jager et al., 2007), however these results need further confirmation. We have shown that in glioblastoma, AMPK promotes mitochondrial metabolism through CREB-dependent activation of NRF2 (Chhipa et al., 2018). PGC1α also controls energy homeostasis and mitochondrial biogenesis by interacting with estrogen-related receptors (ERRs) (Huss et al., 2002; Kamei et al., 2003; Schreiber et al., 2003; Laganiere et al., 2004; Handschin and Spiegelman, 2006; Sonoda et al., 2007; Charest-Marcotte et al., 2010).

Arguments about the role of AMPK in the regulation of glucose metabolism still exist. AMPK activation has been shown to promote expression and cell surface translocation of the glucose transporter GLUT4 through the TF MEF2. On the other hand, PGC1α was shown to function as a coactivator of the MEF2 to regulate GLUT4 expression and surface translocation (Lin et al., 2002; Knight et al., 2003; Holmes et al., 2005; McGee et al., 2006). Whether AMPK directly phosphorylates MEF2 is uncertain, but it has been proposed that AMPK phosphorylates the GLUT4 Enhancer Factor (GEF) to promote its nuclear translocation and interaction with MEF2 (McGee et al., 2006). Another study linked GLUT4 transcription by AMPK through the HDAC5-MEF2 axis. HDAC5 is a histone deacetylase and a transcriptional repressor. It binds to MEF2 and by removing lysine acetyl marks from histones, inhibits MEF2-dependent transcription. In light of these findings, it is worth noting that nuclear AMPK phosphorylates HDAC5 on S259 and 498 disrupting its binding to MEF2 and enabling MEF2-dependent transcription of GLUT4 (McGee et al., 2008).

When overwhelming evidence supports the role of AMPK as a survival kinase during energy crisis, some of the findings about its role in systemic glucose homeostasis during energy crisis seems counterintuitive to its role as a survival kinase. When glucose level drops during fasting, liver glycogenolysis produces glucose; if fasting continues beyond glycogen depletion, gluconeogenesis triggers to provide the much-required glucose for tissue function, particularly the brain and red blood cells. While AMPK phosphorylates CREB1 in muscle to enhance its transcriptional activity, it was shown to reduce CREB1-dependent gluconeogenesis in the liver (Koo et al., 2005). Following depletion of glycogen-derived glucose production, gluconeogenesis is induced in the liver in periods of energy crisis. Glucagon activates cAMP-PKA signaling during which PKA phosphorylates the CREB1 coactivator called transducer of regulated CREB (CRTC2 or TORC2 not mTORC2) causing its nuclear translocation, CREB1 binding and transcription of key gluconeogenic genes such as phosphoenolpyruvate carboxykinase (PEPCK) and glucose-6-phosphatase (G6P). AMPK was shown to phosphorylate CRTC2 on S171 causing cytoplasmic sequestration through 14-3-3 binding and inhibition of gluconeogenesis (Koo et al., 2005). This study showed that AMPK activation even in the presence of cAMP agonists sequestered CREB regulated transcription coactivator 2 (CRTC2) in the cytoplasm and argued that AMPK inhibits gluconeogenesis even in periods of energy crisis which is known to trigger AMPK activation. This phenomenon seems counterintuitive to AMPK’s role as a survival kinase. In fact, the role of CRTC2 in fasting-induced gluconeogenesis and glucose homeostasis has been questioned because fasting-induced glucose homeostasis remained unaffected in CRTC2 knockout mice (Le Lay et al., 2009). Similarly, it is reasonable to assume that AMPK might promote glycogenolysis during starvation. Indeed, initial reports supported this assumption when AMPK was shown to phosphorylate and increase glycogen phosphorylase activity (the rate limiting enzyme for glycogenolysis) (Carling and Hardie, 1989; Young et al., 1996), although this was later disputed by other studies (Viollet et al., 2003a, b; Barnes et al., 2004; Jorgensen et al., 2005). We have conclusively shown that fasting and exercise –induced glycogenolysis in the skeletal muscle (that almost exclusively express β2 and not β1 subunit) is reduced by over 20% in the AMPKβ2 knockout mice (Dasgupta et al., 2012). Together, it seems that AMPK may indeed stimulate glycogenolysis through phosphorylation of glycogen phosphorylase and / or through yet unknown transcriptional control of glycogenolysis.

### AMPK Activity in the Nucleus

Which kinase phosphorylates AMPK in the nucleus or does AMPK get phosphorylated in the cytoplasm before translocating to the nucleus? We and others have shown that AMPK is present in the nucleus. Both α1 and α2 subunits, but the α2 subunit in particular has been found in the nucleus (Salt et al., 1998; Vincent et al., 2001; Leff, 2003; McGee et al., 2003; Kodiha et al., 2007). Unlike the α2, the α1 does not seem to have a nuclear localization signal and its nuclear localization could depend on cytoplasmic-nuclear chaperones. We have shown that active AMPK is present in the nuclei of mouse neural stem cells, and while the regulatory β2 subunit was restricted mainly to the cytoplasm, the β1 subunit was enriched in the nucleus (Dasgupta and Milbrandt, 2009). Examining glioblastoma clinical samples we observed copious amounts of active AMPK both in the cytoplasm and the nucleus (Chhipa et al., 2018). The presence of the AMPK kinase CAMKK2 has been shown in the nucleus of prostate cancer cells (Karacosta et al., 2012), and nuclear AMPKα1 was recently shown to be phosphorylated by CAMKK in HeLa and A549 lung cancer cells (Vara-Ciruelos et al., 2018). Whether LKB1 is present in the nucleus is unknown. Much work is necessary to understand the mechanisms of AMPK activation in the nucleus.

### mTOR Activity in the Nucleus

Accumulating evidence shows the presence of mTOR in the nucleus. mTOR regulates RNA polymerase (Pol)-mediated transcription either directly in the nucleus or indirectly by regulating nuclear translocation of TFs (Giguere, 2018). It is well known that environmental cues (nutrients) regulate the transcriptional activity of Pol I and Pol III. However the mechanism by which nutrients modulate the activity of Pol I and III was ill-defined until the role of mTOR in Pol I and III-mediated transcription of ribosomal DNA and transfer RNA genes was established (Tsang et al., 2010). mTOR associates with the 45s rDNA promoter and 5s rDNA and tRNAs genes, thus, regulating ribosome biogenesis and protein synthesis. mTOR controls the transcriptional activity of Pol I and III by phosphorylating TFs and regulating their activity. For e.g., in *Saccharomyces cerevisiae*, mTORC1 phosphorylates the TF Maf1 through S6K1, thereby controlling Pol-III mediated transcription (Wei et al., 2009). In mammals, mTORC1 phosphorylates TIF1A and UBF, the TFs involved in the formation of Pol I transcription initiation complex (Mayer et al., 2004; Figure 4). Whether cytoplasmic activation of mTORC1 is necessary for its translocation to nucleus or it is activated in the nucleus itself requires further confirmatory studies.

## Concluding Remarks

Since its discovery as a cytosolic metabolic kinase that purified with and inhibited Acetyl Co-A carboxylase (the rate limiting enzyme for long chain fatty acid synthesis) (Lent and Kim, 1982), we have come a long way to have discovered the many facets of AMPK function in cellular and systemic metabolism, regulation of cell cycle, longevity, stress resistance, tumor pathology, circadian rhythm among other functions. Since the generation of the first AMPK subunit knockout animals, seminal studies have provided evidence on its role in glucose and lipid homeostasis in the liver and skeletal muscle and in type II diabetes. The bulk of AMPK research after its discovery as a key negative regulator of mTORC1 was devoted to understand signaling pathways around the AMPK-mTORC1 axis in normal physiology and pathology, particularly cancer. Despite the important findings about AMPK’s direct involvement in gene expression and cell cycle control in the nucleus, frank transcriptional regulation by AMPK beyond signaling has remained a less-explored area. Equally important but also poorly understood is how the individual subunits of AMPK are transcriptionally regulated by cellular and environmental cues. Here, we described an abridged summary of TFs that coordinate transcription of AMPK and mTOR pathway genes. We hope that this preliminary report will engender interest in the field to examine the context-dependent transcriptional regulation of the energy sensing AMPK and biosynthetic mTOR pathway genes.

## Author Contributions

AS and BD wrote the manuscript. KC mined the ENCODE data. All authors contributed to the article and approved the submitted version.

## Conflict of Interest

The authors declare that the research was conducted in the absence of any commercial or financial relationships that could be construed as a potential conflict of interest.

## Footnotes

1 http://jaspar.genreg.net

2 http://genxplain.com/tf_class

3 https://www.encodeproject.org
